# Commonly Used Therapeutics Associated with Changes in Arousal Inhibit GABA_A_R Activation

**DOI:** 10.3390/biom13020365

**Published:** 2023-02-15

**Authors:** Anling Kaplan, Abigail I. Nash, Amanda A. H. Freeman, Lauren G. Lewicki, David B. Rye, Lynn Marie Trotti, Asher L. Brandt, Andrew Jenkins

**Affiliations:** 1 Department of Anesthesiology, Emory University, Atlanta, GA 30322, USA; 2 Department of Medical Affairs, Janssen Scientific Affairs LLC, Titusville, NJ 08560, USA; 3 Center for Human Health, Emory University, Atlanta, GA 30322, USA; 4 School of Pharmacy, University of Saint Joseph, West Hartford, CT 06117, USA; 5 Department of Neurology, Emory University, Atlanta, GA 30322, USA; 6 Department of Chemistry, University of Saint Joseph, West Hartford, CT 06117, USA; 7 Department of Pharmaceutical Sciences, University of Saint Joseph, West Hartford, CT 06117, USA

**Keywords:** gamma-aminobutyric acid, GABA, GABA_A_ receptor, GABA_A_R, negative allosteric modulator, arousal, sleep, idiopathic hypersomnia

## Abstract

GABA_A_ receptor-positive modulators are well-known to induce sedation, sleep, and general anesthesia. Conversely, GABA_A_ receptor negative allosteric modulators (GABA_A_RNAMs) can increase arousal and induce seizures. Motivated by our studies with patients with hypersomnia, and our discovery that two GABA_A_RNAMs can restore the Excitation/Inhibition (E/I) balance in vitro and arousal in vivo, we chose to screen 11 compounds that have been reported to modulate arousal, to see if they shared a GABA-related mechanism. We determined modulation with both conventional and microfluidic patch clamp methods. We found that receptor activation was variably modulated by all 11 compounds: Rifampicin (RIF), Metronidazole (MET), Minocycline (MIN), Erythromycin (ERY), Ofloxacin (OFX), Chloroquine (CQ), Hydroxychloroquine sulfate (HCQ), Flumazenil (FLZ), Pentylenetetrazol (PTZ), (-)-Epigallocatechin Gallate (EGCG), and clarithromycin (CLR). The computational modeling of modulator–receptor interactions predicted drug action at canonical binding sites and novel orphan sites on the receptor. Our findings suggest that multiple avenues of investigation are now open to investigate large and brain-penetrant molecules for the treatment of patients with diminished CNS E/I balance.

## 1. Introduction

Synaptic GABA_A_ receptors (GABA_A_Rs) are the most common fast inhibitory ligand-gated ion channels in the mammalian central nervous system. The receptors are expressed throughout, with differing spatial and temporal patterns [1]. The heterogeneity of subunit composition and anatomic location bestow divergent patterns of inhibition to various brain regions at different times of development [2]. These patterns of inhibition play a key role in E/I balance. Inherited and de novo mutations that alter receptor function lead to changes in the strength or duration of inhibition, and are associated with many neurologic and psychiatric diseases [3].

The endogenous enhancement of receptor function by steroids and naturally occurring peptides leads to impaired arousal [4,5]. The GABA_A_ receptor is also a key therapeutic target for many critical therapeutics and clinically useful drugs. Benzodiazepines, Z-drugs, and many general anesthetics all exert their useful actions by positively modulating the GABA_A_ receptors and mimicking endogenous modulation, resulting in sedation and unconsciousness [6].

Motivated by our discoveries that pathological sleep in humans can be reversed with a benzodiazepine antagonist and a macrolide antibiotic [7,8,9,10], we sought to screen commonly used therapeutics that have previously been associated with sleep disruption. Our goal was to discover GABA_A_RNAM activity that may be leveraged to improve arousal in patients with diseases associated with excessive daytime sleepiness.

## 2. Compounds Tested and Rationale

The molecules tested in this study have previously been associated with neurologic and psychological alterations, and changes in sleep. Rifampicin (RIF) is an antibiotic whose side effect profile includes sedation. Metronidazole (MET) is used to treat anaerobic and protozoal infections, and it is administered orally with a bioavailability >90%. MET reaches 60–100% of plasma concentrations in the CNS, and the pharmacodynamics show activity for 12–24 h after 1 g [11]. MET also has been shown to reduce slow wave sleep (SWS) and increase sleep latency [12]. Minocycline (MIN) is a tetracycline antibiotic that crosses the blood–brain barrier (BBB), and it is used to treat bacterial infections such as pneumonia and respiratory tract infections. MIN has been shown to have anti-inflammatory and neuroprotective functions, and it has been proposed to have a role in treating anxiety and depression [13]. MIN also inhibits protein synthesis, and it appears to reduce SWS [14]. Erythromycin (ERY) is a macrolide antibiotic in the same class as clarithromycin, and it is used to treat and to prevent a wide variety of bacterial infections. In a pilot study in patients suffering from chronic fatigue syndrome, 400 mg ERY altered sleep, possibly due to changes in gut microbiota [15].

Quinolone antibiotics are known to interact directly with the GABA_A_ receptor. Ofloxacin (OFX) is a fluoroquinolone antibiotic that has been shown to induce hypomania and decrease the need for sleep [16]. OFX shortens sleep time in mice, and it has a dose-dependent psychostimulant effect [17]. OFX has been shown to inhibit the GABA binding in isolated rat neurons in a concentration-dependent manner [18]. Chloroquine (CQ) is used to treat malaria; it has been shown to interact with Cys-loop receptors, and it competitively binds to GABA_A_Rs [19]. Hydroxychloroquine sulfate (HCQ) is an analog of CQ; it is also used to treat malaria, and it can also act as an anti-inflammatory agent [20]. HCQ is commonly used to treat rheumatoid arthritis, and it is safe for long-term use and has been reported to improve the quality of sleep in patients with primary Sjögren’s syndrome who experience a decreased quality of life due to poor sleep quality and excessive daytime sleepiness [21].

Two of the drugs tested here have been used successfully in clinical practice and clinical trials for Idiopathic Hypersomnia (IH). Flumazenil (FLZ) is a GABA_A_ receptor antagonist that competitively inhibits activity at the benzodiazepine (BZD) site of the GABA_A_ receptor complex and is FDA approved for the reversal of benzodiazepine-induced sedation. It is believed to antagonize the sedative hypnotic actions of BZDs at the γα subunit interface on GABA_A_ receptors [7]. Our lab has previously shown that flumazenil acts as a NAM at the GABA_A_ receptor when activated in the presence of GABA and CSF from hypersomnolent patients. Flumazenil has also had success in the clinical treatment of patients with IH [7,22]. Clarithromycin has been shown to reduce self-reported sleepiness and other disease symptoms in a pilot clinical trial, including participants with IH and other related disorders. Pentylenetetrazol (PTZ) has been previously reported as a GABA antagonist, and it has a long history of clinical use to treat overdoses and psychosis [23]. PTZ has also been shown to be effective in animal models of Down syndrome [24,25,26], where sleep problems such as excessive daytime sleepiness are common [27]. (-)-Epigallocatechin Gallate (EGCG) is a flavonoid which has been reported to interact with GABA_A_ receptors, and it can either act as a positive or negative allosteric modulator [28]. It has been reported that low caffeine green tea improves sleep quality and reduces fatigue, based on the fact that caffeine and EGCG suppress the anti-stress effects of theanine [29].

## 3. Methods

### 3.1. Molecular Biology and Cell Culture

Human GABA_A_ receptor cDNAs (α1β2γ2s) were subcloned into the pcDNA 3.1 expression vector, and they were subsequently transfected into HEK293 cells using standard methods. Forty-eight hours later, cells were exposed to individual GABA_A_RNAM solutions with simultaneous exposure to GABA. GABA_A_RNAMs were obtained from Sigma-Aldrich, and the solutions were prepared with a saline solution to 1–300 µM concentrations. For rifampicin, 0.5% DMSO was also present in solution for solubility. Stable cell lines were generated and handled as previously described [30].

### 3.2. Conventional Patch Clamp

Whole-cell recordings—Wild type GABA_A_ receptors were characterized via whole-cell, voltage-clamp electrophysiology at room temperature 36–72 h after transfection. Patch pipettes were fabricated from thin-walled borosilicate glass (TW150F-4, World Precision Instruments, Inc., Sarasota, FL, USA) using a horizontal puller (P-97, Sutter Instrument Co., Novato, CA, USA). The resistances of the patch pipettes were 2–5 megaohms when filled with an intracellular solution containing 120 mM KCl, 2 mM MgCl_2_, 10 mM EGTA, and 10 mM HEPES, adjusted to pH 7.2 with NaOH. Cells were perfused continuously with an extracellular solution containing 160 mM NaCl, 10 mM HEPES, 6 mM D-glucose, 3 mM KCl, 1 mM MgCl_2_, and 1.5 mM CaCl_2_, adjusted to pH 7.4. Two 10-channel infusion pumps (KD Scientific) and a rapid solution exchanger (Biologic) were used to apply extracellular saline to the cells. Whole-cell currents were recorded at −60 mV, digitized at 100 Hz, and filtered at 50 Hz with a MultiClamp 700B amplifier and a DigiData 1322A interface (Molecular Devices) for offline analysis using bespoke scripts written in MATLAB. The solution changer was driven by protocols written in pClamp 9.2 (Molecular Devices).

### 3.3. Microfluidic Patch Clamp

Microfluidic electrophysiology: Intracellular saline solutions contained 145 CsCl, 2 CaCl_2_, 2 mM MgCl_2_, 10 mM EGTA, and 10 mM HEPES. Extracellular saline solutions contained 140 mM NaCl, 5 mM KCl, 2 mM CaCl_2_, 1 mM MgCl_2_, 10 mM HEPES, and 10 mM D-glucose. Both salines were adjusted to pH 7.4 (330 mOsm) and were 0.2 µm filter sterilized. The composition was based on solutions designed for long duration HEK293 biophysics studies [31]. HEK293 cells stably expressing mouse α1β2γ2L receptors [30] were dissociated with detachin (Genlantis, San Diego, CA, USA), resuspended in extracellular saline (2.5 × 10^6^ cells/mL), and agitated prior to loading into 384-well Fluxion ensemble plates. In our hands, these cells can be passaged 3–40 times and still produce reproducible responses to GABA for over 2 years. In addition, we have found that electrophysiology experiments using 30 s episodes are stable and free of rundown for over 45 min. GABA concentration response relationships indicate that 100 µM GABA elicited >90 % of the maximal response.

**Figure 1:**
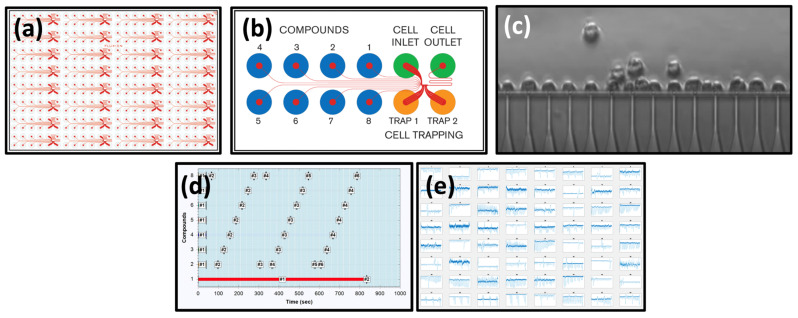
Microfluidic experimental layout. (**a**) A 384-well HT Mercury plate layout showing 32 repeats of the same perfusion system. The orange color depicts the 12 wells and the microfluidic channels connecting them. (**b**) Single experimental pattern and microfluidic channels. Compounds are loaded into wells 1–8 (blue), cells are loaded into the inlet and intracellular solutions in the 2 traps well (orange). (**c**) Fourteen HEK293 cells trapped prior to whole cell seal formation. (**d**) Eight compounds and nine dose response protocols. (**e**) Representative data file with 192 dose responses collected in 15 min.

Cells were patched using an Ionflux Mercury microfluidic automated patch clamp system [32,33] (Fluxion Biosciences, Oakland, CA, USA). Each experiment ran on a disposable 384-well plate with microfluidic channels printed into an ionized polydimethylsiloxane base. Each plate was divided into 4 zones, each containing 8 repeats of the same experimental pattern (see Figure 1). Each pattern comprised 8 compound wells containing the ligands/modulator combinations to be used in the experiment. Connected to these by a manifold perfusion chamber was an inlet well containing the cell suspension, an outlet well for discarded perfusate, and 2 trapping wells. Both trapping wells contained intracellular solution and were connected to the manifold via 20 1 µm diameter holes for cell attachment (each pattern can patch up to 40 cells simultaneously). Intracellular electrodes were placed in the 2 trapping wells, and the reference electrode in the waste well. Electrodes were connected to 64 conventional low-noise resistive feedback amplifiers. After cleaning, the plates were loaded with up to 100 µL of experimental solution or cell suspension per well. Each plate was loaded into the Fluxion Ionflux Mercury device, and a bespoke script primed the experimental fluidic channels and manifold, and the traps and sealed cells for voltage clamp measurements, and finally, controlled solution application/concentration jumps prior to saving the data collected. Agonist applications were repeated in triplicate (see Supplemental Figure S1). The current data were recorded at 10 kHz and stored for offline analysis. MATLAB scripts were used to measure the baseline leak current and peak response for each episode of data (typically 2000–4000/experiment). The matrix of peaks was expressed as a percentage of the response to a reference concentration. For the agonist concentration–response experiments, this was the maximum response. For the modulator concentration–response experiments, this was the EC_10_ agonist response.

### 3.4. In Silico Modeling Methods

The published cryo-EM structure (PDB: 6DW0) of the GABA A receptor in complex with GABA was used as a starting template [34]. The 6DW0 was prepared using a protein preparation wizard (Schrödinger, New York, NY, USA), where the bond orders were assigned and the structure was minimized. Each of the structures: chloroquine, erythromycin, metronidazole, minocycline, ofloxacin, (-)-epigallocatechin gallate, flumazenil, hydroxychloroquine, pentylenetetrazol, clarithromycin, and rifampicin, were built and optimized in a Spartan ‘20 Parallel Suite (Wavefunction, Irvine, CA, USA). Tautomers were generated for each ligand, and the dominant ionization states of acidic and basic functional groups were used, assuming pH 7.4 (Schrödinger, New York, NY, USA). QikProp was used to calculate the ADME properties of the 11 ligands tested (Schrödinger, New York, NY, USA). A receptor grid generator was used to place 10 Å × 10 Å × 10  Å boxes covering the whole receptor (Schrödinger, New York, NY, USA). All 11 ligands were run for each of the grid boxes generated in Glide SP, using flexible docking with enhanced conformational sampling by four-fold, and an expanded sampling for the selection of initial poses (Schrödinger, New York, NY, USA). The binding site for each ligand was chosen based on the most negative glide score. Erythromycin was not found to bind to any of the sites, and thus it was not carried forward. For each ligand–receptor complex, a minimization was run to reduce steric clashing between the ligand–receptor complex (Schrödinger, New York, NY, USA) using the VGSB solvation model. MMGBSA was run on each ligand–receptor complex to determine their relative affinities.

## 4. Results

### 4.1. Electrophysiology

**Figure 2:**
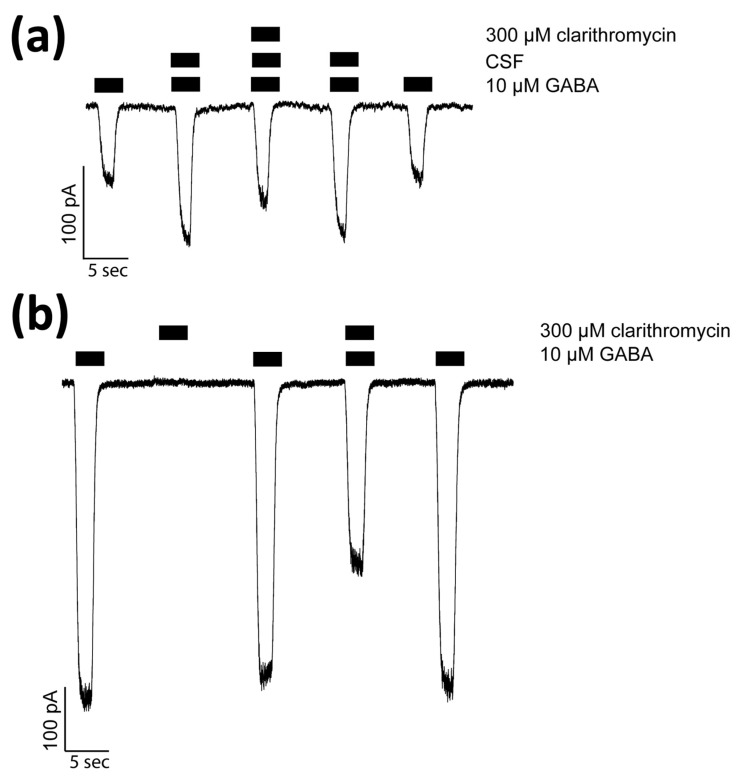
Clarithromycin inhibits CSF-mediated GABA receptor potentiation and activation. Whole cell voltage clamp recordings from HEK293 cells expressing α1β2γ2s GABA_A_Rs. (**a**) CSF from a patient diagnosed with Idiopathic Hypersomnia reversibly enhances/potentiates currents activated by 10 μM GABA (EC_10_). The potentiation is reduced in the presence of 300 μM clarithromycin. (**b**) Clarithromycin has no intrinsic efficacy at GABA_A_ receptor, but reversibly inhibits receptor activation. Bars above the current traces indicate the duration of agonist/modulator application. Calibration bars indicate current amplitude and duration in pA and seconds.

**Figure 3:**
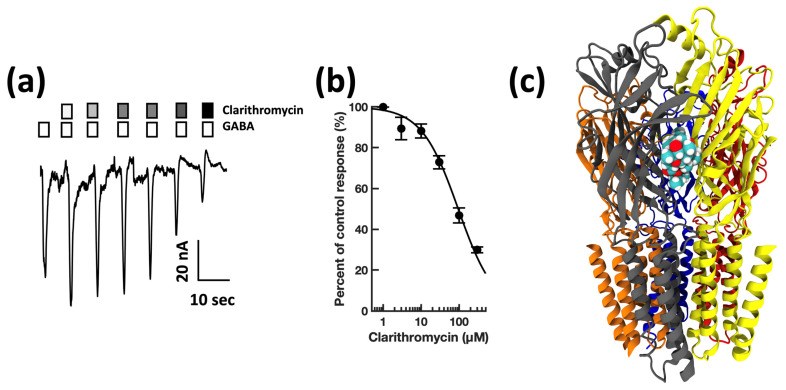
Clarithromycin inhibits GABA_A_ receptor activation in a concentration-dependent manner, and preferentially docks at the α + β-Interface in a pocket defined by canonical loops A-F. (**a**) Representative microfluidic ensemble whole cell recording from HEK293 cells expressing rat α1β2γ2s GABA_A_ receptors. Open bars above the current trace indicate the duration of GABA application. Shaded bars indicate the duration of clarithromycin application. Darker shading indicates increased concentration. The concentrations applied were 1, 3, 10, 30, 100, and 300 μM clarithromycin. Calibration bars indicate the current amplitude and duration in nA and seconds. (**b**) Clarithromycin concentration response relationship for GABA receptor modulation. Each point is plotted as mean ± standard error of the mean for 20–24 determinations. (**c**) The clarithromycin binding pocket is predicted to be defined by the α (loops A and C, and the β1β2 turn) and β (Loops D, F) subunits. Color key: α subunit (red and grey), β subunit (yellow and orange), γ subunit (blue).

**Figure 4:**
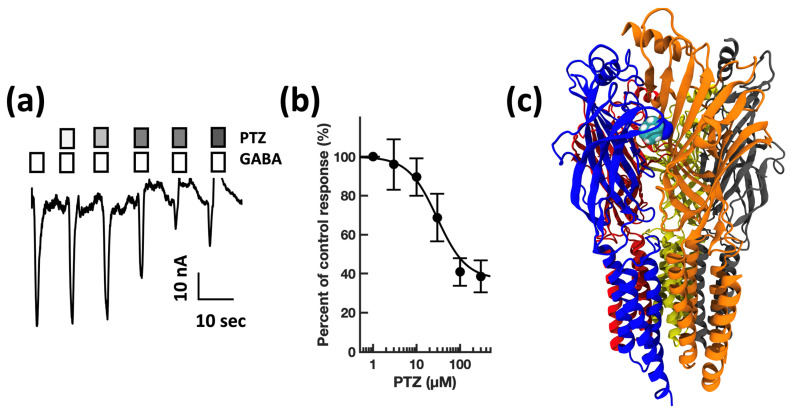
Pentylenetetrazole inhibits GABA_A_ receptor activation in a concentration-dependent manner, and preferentially docks at the γ + β Interface in a pocket defined by canonical loops A–E. (**a**) Representative microfluidic ensemble whole cell recording from HEK293 cells expressing rat α1β2γ2s GABA_A_ receptors. Open bars above the current trace indicate the duration of GABA application. Shaded bars indicate the duration of pentylenetetrazole application. Darker shading indicates increased concentration. The concentrations applied were 1, 3, 10, 30, and 100 μM pentylenetetrazole. Calibration bars indicate current amplitude, and duration in nA and seconds. (**b**) Pentylenetetrazole concentration response relationship for GABA receptor modulation. Each point is plotted as mean ± standard error of the mean for 20–24 determinations. (**c**) The pentylenetetrazole binding pocket is predicted to be defined by the γ (loops D and E) and β (Loops A, B, and C) subunits. Color key: α subunit (red and grey), β subunit (yellow and orange), γ subunit (blue).

**Figure 5:**
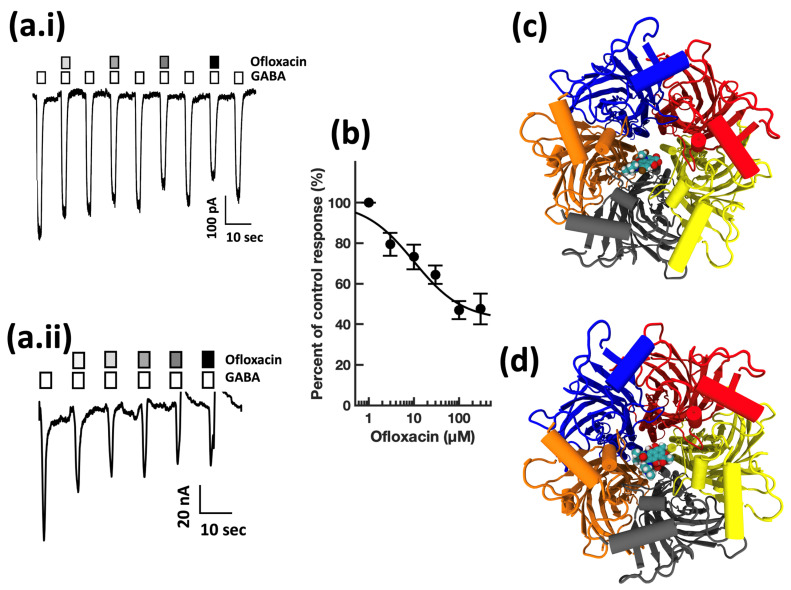
Ofloxacin inhibits GABA_A_ receptor activation in a concentration-dependent manner, and preferentially docks in the channel lumen at a location shared by rifampicin. (**a.i**) Conventional single electrode whole cell voltage clamp recording from a HEK293 cell expressing human α1β2γ2s GABA_A_Rs. Open bars above the current trace indicate the duration of GABA application. Shaded bars indicate the duration of ofloxacin application. Darker shading indicates increased concentration. The concentrations applied were 1, 3, 10, 30 μM ofloxacin. Calibration bars indicate current amplitude and duration in pA and seconds. (**a.ii**) Representative microfluidic ensemble whole cell recording from HEK293 cells expressing rat α1β2γ2s GABA_A_ receptors. Open bars above the current trace indicate the duration of GABA application. Shaded bars indicate the duration of ofloxacin application. Darker shading indicates increased concentration. The concentrations applied were 3, 10, 30, 100, and 300 μM ofloxacin. Calibration bars indicate current amplitude, and duration in nA and seconds. (**b**) Ofloxacin concentration response relationship for GABA receptor modulation. Each point is plotted as mean ± standard error of the mean for 20–24 determinations. (**c**) The ofloxacin binding pocket is predicted to be defined by the lumen-facing residues of the BAE chains (βαβ subunits). (**d**) The rifampicin binding pocket is predicted to be defined by lumen-facing residues of the BAED chains (βαβγ subunits). Color key: α subunit (red and grey), β subunit (yellow and orange), γ subunit (blue).

**Figure 6:**
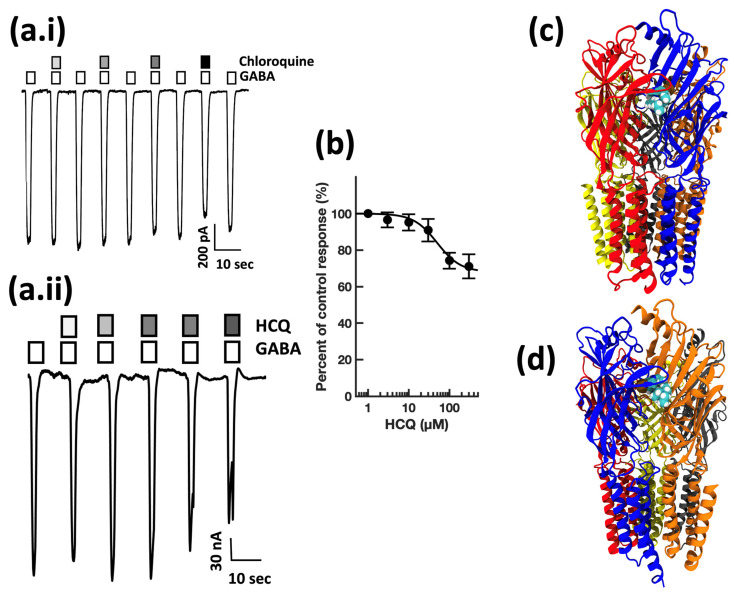
Chloroquine and hydroxychloroquine inhibit GABA_A_ receptor activation in a concentration-dependent manner, and dock to pockets on the γ subunit. (**a.i**) Conventional single electrode whole cell voltage clamp recording from a HEK293 cell expressing human α1β2γ2s GABA_A_Rs. Open bars above the current trace indicate the duration of GABA application. Shaded bars indicate the duration of chloroquine application. Darker shading indicates increased concentration. The concentrations applied were 1, 3, 10, and 30 μM chloroquine. Calibration bars indicate current amplitude, and duration in pA and seconds. (**a.ii**) Representative microfluidic ensemble whole cell recording from HEK293 cells expressing rat α1β2γ2s GABA_A_ receptors. Open bars above the current trace indicate the duration of GABA application. Shaded bars indicate the duration of hydroxychloroquine application. Darker shading indicates increased concentration. The concentrations applied were 3, 10, 30, 100, and 300 μM hydroxychloroquine. Calibration bars indicate current amplitude and duration in nA and seconds. (**b**) Hydroxychloroquine concentration response relationship for GABA receptor modulation. Each point is plotted as mean ± standard error of the mean for 20–24 determinations. (**c**) The chloroquine binding pocket is predicted to be defined by the α (Loops A, B, and C) and γ (Loops D, E, and F) subunits, overlapping with the canonical benzodiazepine binding site. (**d**) The hydroxychloroquine binding pocket is predicted to be defined by the β (loops D, E, and F) and γ (loops A, B, and C) subunits. Color key: α subunit (red and grey), β subunit (yellow and orange), γ subunit (blue).

**Figure 7:**
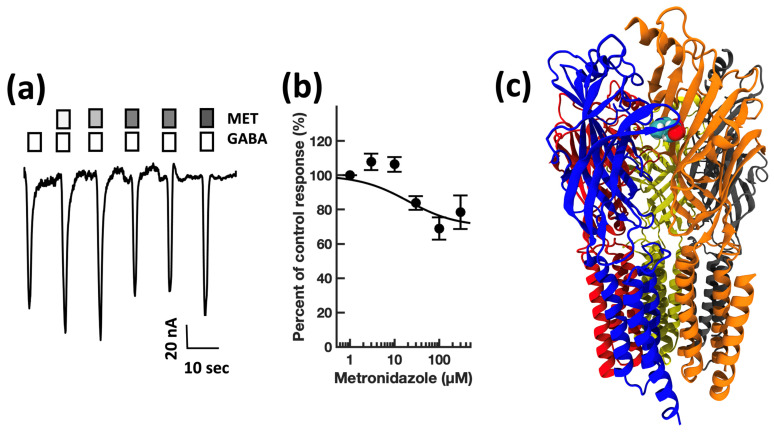
Metronidazole modestly inhibits GABA_A_ receptor activation in a concentration-dependent manner, and preferentially docks at the γ+β—Interface in a pocket defined by canonical loops A–E. (**a**) Representative microfluidic ensemble whole cell recording from HEK293 cells expressing rat α1β2γ2s GABA_A_ receptors. Open bars above the current trace indicate the duration of GABA application. Shaded bars indicate the duration of metronidazole application. Darker shading indicates increased concentration. The concentrations applied were 3, 10, 30, 100, and 300 μM metronidazole. Calibration bars indicate the current amplitude and duration in nA and seconds. (**b**) Metronidazole concentration response relationship for GABA receptor modulation. Each point is plotted as mean ± standard error of the mean, for 20–24 determinations. (**c**) The metronidazole binding pocket is predicted to be defined by the β (loops D, E, and F) and γ (Loops B and C) subunits. Color key: α subunit (red and grey), β subunit (yellow and orange), γ subunit (blue).

**Figure 8:**
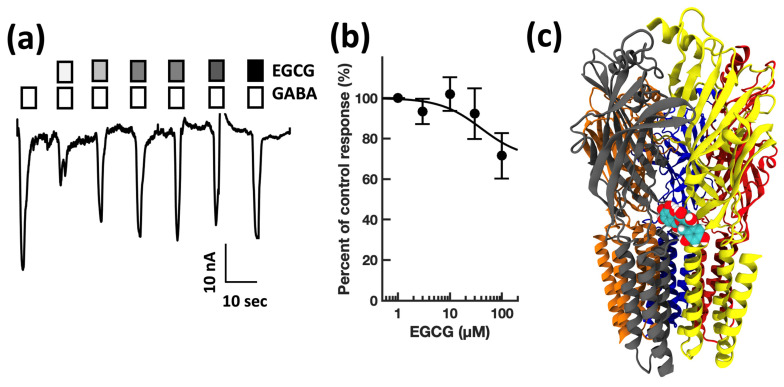
EGCG modestly inhibits GABA_A_ receptor activation in a concentration-dependent manner, and preferentially docks at the α + β Interface in a pocket defined by a TM23 linker, a cys-loop, and one loop F. (**a**) Representative microfluidic ensemble whole cell recording from HEK293 cells expressing rat α1β2γ2s GABA_A_ receptors. Open bars above the current trace indicate the duration of GABA application. Shaded bars indicate the duration of EGCG application. Darker shading indicates increased concentration. The concentrations applied were 1, 3, 10, 30, 100, and 300 μM EGCG. Calibration bars indicate current amplitude and duration in nA and seconds. (**b**) EGCG concentration response relationship for GABA receptor modulation. Each point is plotted as mean ± standard error of the mean for 20–24 determinations. (**c**) The EGCG binding pocket is predicted to be defined by the α (TM23 linker and the cys-loop) and β (Loop F) subunits. Color key: α subunit (red and grey), β subunit (yellow and orange), γ subunit (blue).

First, guided by our clinical experience, we investigated whether clarithromycin could reverse the CSF-induced enhancement of the GABA receptor function, as we have previously shown with flumazenil [7]. Using a conventional single electrode patch clamp, we found that CSF from hypersomnolent patients enhanced GABA by 89 ± 11% (*n* = 6 cells). Clarithromycin reversed this potentiation by 71 ± 13% (*n* = 6 cells, see Figure 2a). However, unlike flumazenil, we observed that clarithromycin was also able to inhibit GABA_A_ receptor activation (Figure 2b), revealing that unlike flumazenil’s benzodiazepine antagonist actions, clarithromycin acts as a GABA_A_ receptor negative allosteric modulator or a GABA antagonist. Next, we chose to replicate this finding using a high throughput microfluidic patch clamp assay. We found this technique readily reproduced our previous finding, validating it as a suitable replacement for the traditional low-throughput technique. Using the ion flux system, we determined that clarithromycin reversibly inhibited GABA_A_ receptor activation in a concentration-dependent manner up to 300 µM (see Figure 3). Next, we sought to further validate our microfluidic method using the well-established GABA_A_RNAM pentylenetetrazole (PTZ). Again, we were able to demonstrate that this method was well-suited to measuring GABA_A_ receptor modulation, and reproduced previous findings, showing that PTZ blocked GABA_A_R activation in a concentration-dependent manner (see Figure 4 and Table 1). Next, we determined the effect of ofloxacin on GABA_A_R activation using both electrophysiologic techniques. In both cases, we found that ofloxacin produced a modest and incomplete block of receptor activation (see Figure 5 and Table 1). Similarly, we found that chloroquine and hydroxychloroqine exhibited a modest and incomplete block of receptor activation at the highest concentrations used (see Figure 6 and Table 1). Finally, using only the microfluidic method, we similarly found that metronidazole and EGCG produced a modest inhibition of GABA activation (see Figure 7 and Figure 8 and Table 1).

### 4.2. Modeling

First, for our docking studies, we did not preselect any candidate binding sites or pockets, nor did we bias our results by choosing specific binding sites for each compound. Instead, we used the comprehensive method described above to find the best score for each ligand–receptor pair throughout the whole receptor. To validate our methodology, we first docked flumazenil to the 6DW0.PDB GABA_A_ receptor structure. We were pleased when our method predicted that flumazenil binds to the α^+^γ^−^ canonical benzodiazepine binding site, where it is known to interact [45], validating our approach (see Supplemental Figure S2).

**Figure 9:**
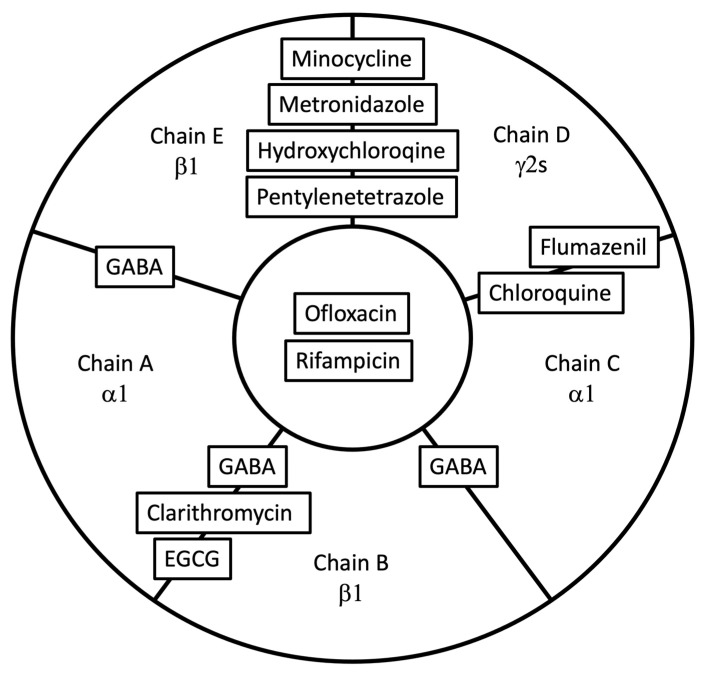
Cartoon of the predicted binding pockets of the compounds used in this study on the GABA_A_R extracellular surface, as viewed from a synapse. No modulators were predicted to act at the 2 canonical (αβ) GABA binding sites. Clarithromycin and EGCG are predicted to act at the βα GABA binding site. Flumazenil and chloroquine both docked at the canonical benzodiazepine γα binding site. Minocycline (data not shown), metronidazol, hydroxychloroquine, and pentylenetetrazole were all predicted to interact with the orphan βγ binding site. Finally, ofloxacin and rifampicin were docked in the extracellular lumen.

Next, our docking studies predicted that the 8/9 of molecules under investigation could bind one of four distinct binding pockets in the 6DW0.pdb model of the rat α1β1γ2s receptor. The only exception was erythromycin, which failed to interact with the model. We found that none of the compounds located into either of the two canonical GABA β^+^α^−^ binding sites, or any of the transmembrane anesthetic binding pockets. Our docking studies predicted that chloroquine, like flumazenil, can locate in the α^+^γ^−^ benzodiazepine binding site (see Figure 6c). Next, we found that ofloxacin and rifampicin can bind to the central axial extracellular lumen between the five subunits (see Figure 5c,d). Clarithromycin preferentially docks to the α^+^β^−^ cavity that until recently was thought to be an orphan homologue of the two β^+^α^−^ GABA binding sites (see Figure 3c). However, this view has recently been challenged, and this site has been proposed to be a third GABA binding site [34]. We found that EGCG also docked at this interface, but closer to the transmembrane region where it can interact with residues in the cys-loop and the TM23 linker. Finally, the orphan binding pocket at the γ^+^β^−^ interface is predicted to interact with MIN, HCQ, MET, and PTZ (see Figure 6d, Figure 7c and Figure 4c). This latter finding is particularly interesting, since despite its long clinical use and known interactions with GABA_A_ receptors, no binding site has been proposed for PTZ. The in silico modeling parameters determined in this study are shown in Table 2, and the different docking site locations are summarized in Figure 9.

## 5. Discussion

**Figure 10:**
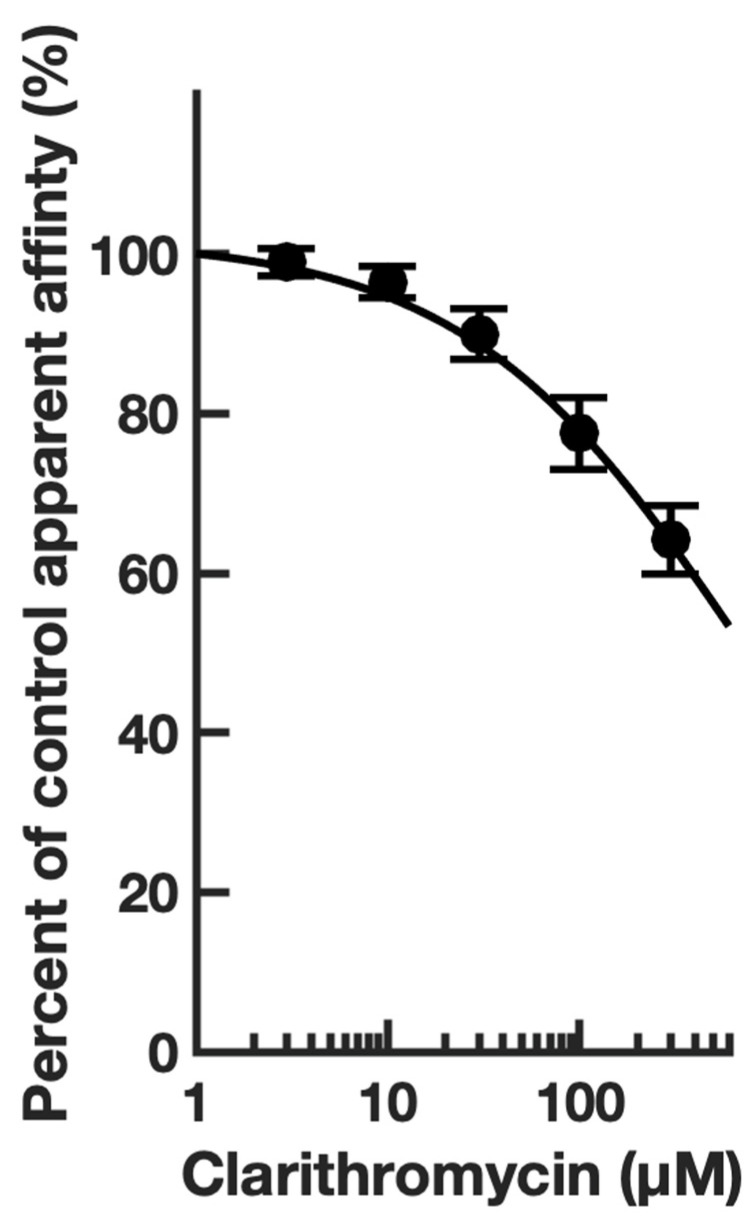
Increasing clarithromycin concentration raises the GABA EC_50_ exponentially. The GABA EC_50_ was determined at 6 different clarithromycin concentrations (0–300 μM). The increase in EC_50_ for each clarithromycin concentration is plotted as a decrease in apparent affinity.

Our conventional and microfluidic electrophysiology experiments demonstrated that clarithromycin reduces GABA_A_R activation and CSF-mediated potentiation. Our modeling studies suggest that it interacts with a distinct pocket on the receptor, previously thought to be an orphan site in GABA_A_Rs. Recently, the α^+^β^−^ interface has been shown to contain electron density at saturating GABA concentrations that could order water, but more likely, a natural ligand [34]. If clarithromycin also binds at this location, it could act as a competitive GABA antagonist. If this is the case, we hypothesize that increasing the concentrations of clarithromycin would reduce the apparent affinity of the receptor for GABA limitlessly (or until the clarithromycin concentration reaches its solubility limit). Conversely, noncompetitive inhibition would produce a limited reduction in affinity, with receptor activation tending towards a finite GABA EC_50_. To test this hypothesis, we constructed GABA concentration–function relationships in the presence of 0–300 μM clarithromycin. The effect of clarithromycin concentration on GABA EC_50_ is shown in Figure 10. Even as the clarithromycin concentration tended towards its aqueous solubility limit, the GABA EC_50_ continued to increase. This reduction in apparent affinity suggests a competitive mechanism for clarithromycin, and support for the hypothesis that the α^+^β^−^ pocket constitutes a third GABA binding site.

Our data suggest that the chalate EGCG inhibits GABA activation and shares the α^+^β^−^ GABA-binding pocket. Campbell et al. have also shown that EGCG blocks GABA activation [28]. In their detailed study, they show that the block is underpinned by an increase in the GABA EC_50_. Taking these observations together, we hypothesize that the α^+^β^−^ interface contains a functional GABA binding site that can be targeted by the competitive antagonists clarithromycin and EGCG. The GABA antagonist actions of these compounds are consistent with their use in humans, where they are both associated with increased arousal [9,10,29].

The convulsant pentylenetetrazole is a well-known GABA_A_R blocker [46]. Its site and mechanism of action are equivocal. Early studies suggested an overlapping binding site with benzodiazepines [47]. This view changed to the general consensus that pentylenetetrazol is a channel blocker [48,49], and that it shares a transmembrane binding site with picrotoxin, which has now been resolved using cryo-electron microscopy [45]. However, the PTZ binding site consensus has been elegantly challenged [50], suggesting while there may be some overlap between the mechanisms of PTX and PTZ, the sites are distinct. Our findings support this latter finding. Using microfluidic patch clamp, we were able to demonstrate that PTZ was an effective inhibitor of GABA activation, and our modeling studies suggest that it does not prefer binding in the M2 2′-9′ region of the channel, instead docking favorably in the orphan γ^+^β^−^ binding site. Interestingly, our structural models suggest that this site can also bind to the modest inhibitors metronidazole and hydroxychloroqine, and the irreversible blocker minocycline (data not shown).

Our findings have broader implication for the study of neuropharmacology and the treatment of patients with arousal disorders. In rat pyramidal neurons, clarithromycin has been shown to induce hyperexcitability, increase the firing rate, and reduce the amplitude of spontaneous miniature inhibitory postsynaptic GABAergic currents [51]. Our data strongly support these findings. Together, our work and previous studies suggest a mechanism of action for clarithromycin reversing sleepiness in patients suffering from idiopathic hypersomnia. In this population, clarithromycin has been shown to improve subjective measures of sleep [9]. A recent meta-analysis of the available clinical data continues to favor the use of clarithromycin in the treatment of hypersomnia in the majority of cases [52].

Our clinical experience led us to investigate clarithromycin as a potential treatment for idiopathic hypersomnia, and as a potential novel GABA_A_RNAM. Since clarithromycin is not well-tolerated by many patients (the side effects include metallic taste, nausea, and headache), we sought to test other macrolides and antibiotics to see if they may also be useful in treating hypersomnia via a GABAergic mechanism. We were further motivated in this effort by the previous research into GABA_A_R modulation by antibiotics from several drug classes, since many therapeutics used to treat infections are associated with disturbances in arousal [53,54]. For example, the β-lactam penicillin, is known to be a GABA_A_R channel blocker [55], and quinolone antibiotics inhibit GABA receptor activation [56]. We were therefore hopeful that other macrolides and/or tetracyclines, ansamycins, etc., may demonstrate activity at the GABA_A_ receptor, and may represent additional therapeutic options for patients with poorly controlled excessive daytime sleepiness.

Our results with the quinolones chloroquine and hydroxychloroqine are consistent with this class of compounds having modest action at GABA_A_Rs. However, the prediction that the two chemically similar compounds prefer different binding pockets was not anticipated. Our studies indicate that the β^−^γ^+^ pocket can accommodate a diverse array of molecules, including hydroxychloroquine, yet chloroquine appears to prefer docking to the γ^−^α^+^ pocket.

The promiscuous nature of the interactions predicted here is interesting and has been explored previously [57]. GABA_A_Rs contain a well-characterized agonist, GABAPAM and GABA_A_RNAM binding sites. The GABA and benzodiazepine agonist sites are found on either side of the alpha subunits (α^−^ and α^+^, respectively) while low-affinity GABAPAMs are thought to act at cavities between the helical transmembrane segments [45,58,59]. GABA_A_RNAMs seem to predominantly locate to the channel lumen. Our findings, while in part supporting this simple model, suggest that multiple sites, some novel, may play a role in receptor modulation and the side effects produced by the drugs studies here.

The clinical relevance of our findings are notable. With one exception (EGCG), the CNS concentration of the modulator is at least 37% of the GABA IC_50_. Put another way, during normal therapeutic use, the compounds investigated here will have a significant effect on receptor activation in the CNS. Given the safety profile of these compounds, our findings suggest that some of these compounds might be useful in treating excessive sleepiness.

Finally, our studies have demonstrated that microfluidic patch clamp methods are a suitable substitute to conventional methods for screening GABA_A_ receptor modulators. We found that the microfluidic approach had several advantages over conventional methods. First, the apparatus is straightforward to use, and unlike conventional methods, it does not require prolonged training. Second, the miniaturized set-up means that drug or biological fluid use is reduced by 2–10-fold per data point. This is especially useful if screening small volumes of human samples, expensive therapeutics, or screening difficult-to-synthesize compounds. In fact, while each determination may need only 50 uL of drug to set up the assay, after the experiment, 90% may be reclaimed for subsequent analysis. Third, the 384-well plate format facilitates the collection of large datasets (2000–4000 data episodes/hour). Finally, since the ensemble plate allows us to record from multiple cells simultaneously, this reduces the impact of cell–cell EC_50_ variability. This is especially useful when studying allosteric modulators where strict adherence to a fixed reference of agonist is a critical part of the experimental design.

## 6. Conclusions

The compounds tested in this study are structurally diverse, yet they all modulate GABA_A_ receptor activation and can impair arousal in humans. From a structure–activity perspective, this may seem counterintuitive. Our modeling data provide an insight into this paradox, and show how the GABA_A_ receptor can act as a nexus for these compounds by binding them at multiple sites that in turn, impair receptor activation, translating the binding of disparate molecular structures into changes in a common pathway.

Guided by the side effects associated with the therapeutics studies here, we have developed a pipeline of clinically safe compounds that may be useful in treating the symptoms of pathological sleepiness. These findings may benefit patients diagnosed with Idiopathic Hypersomnia, Type 1 Myotonic Dystrophy, or Kleine-Levin Syndrome, who experience excessive daytime sleepiness that is not controlled with conventional stimulants. Future work in human subjects and with human CSF samples are needed to determine the mechanisms of action and the therapeutic potential of these compounds.

## Figures and Tables

**Table 1:** Clinical and physiologic parameters for 10 compounds studied with GABA_A_ receptors in this study. Concentrations are given in μM. Data are mean ± standard error of the mean for 20–24 determinations. [CNS] denotes concentration of drug in the CNS. I_max_(%) denotes maximum inhibition.

Drug	Class	Use	[CNS]	GABA EC_50_	I_max_ (%)	n_H_
EGCG	polyphenol	dietary supplement	0.18 [35]	31 ± 4	22 ± 8	1.1
Chloroquine	aminoquinolone	antimalarial	1.60 [36]			
Clarithromycin	macrolide	antibiotic	10 [37]	20 ± 1	85 ± 8	1
Erythromycin	macrolide	antibiotic	0.68 [38]			
Hydroxychloroquine	aminoquinolone	antimalarial	50 [39]	40 ± 8	29 ± 4	1.4
Metronidazole	nitroimidazole	Antibiotic/protozoal	7010 [40]	50 ± 11	24 ± 9	0.8
Minocycline	tetracycline	antibiotic	1.1 [41]			
Ofloxacin	quinolone	antibiotic	5.6 [42]	15 ± 4	61 ± 7	1
pentylenetetrazole	azepine	stimulant/convulsant	330 [43]	10 ± 3	68 ± 5	1.3
Rifampin	ansamycin	antibiotic	3.60 [44]			

**Table 2:** In silico modeling parameters for docking 11 compounds to the 6DW0.pdb GABA_A_ receptor structure.

Compound	Glide Score	MMGBSA	QPlogPo/w	QPlogkhsa	CNS
Chloroquine	−9.528	−69.04	4.653	0.629	1
Erythromycin	N/A	N/A	2.558	−0.060	−1
Metronidazole	−6.505	−38.49	−0.019	−0.688	−2
Minocycline	−6.690	−47.42	0.323	0.178	−2
Ofloxacin	−4.224	−30.44	−0.388	−0.431	0
(-)-Epigallocatechin gallate	−10.319	−65.68	−0.275	−0.447	−2
Flumazenil	−9.324	−44.59	1.956	−0.442	0
Hydroxychloroquine	−10.956	−65.90	3.564	0.207	1
Pentylenetetrazol	−6.954	−33.85	0.555	−0.658	0
Clarithromycin	−3.271	−34.09	3.090	0.058	0
Rifampicin	−3.052	−41.18	3.302	−0.181	−2

## Data Availability

The data presented in this study are available on request from the corresponding author.

## Supplementary Materials

The following supporting information can be downloaded at: https://www.mdpi.com/article/10.3390/biom13020365/s1, Figure S1: Exemplar Ionflux Mercury current recording. Figure S2: Predicted binding pose for flumazenil. Figure S3: Detailed view of predicted binding poses for 10 compounds studied.
