# Supplementary material for: Commonly Used Therapeutics Associated with Changes in Arousal Inhibit GABAAR Activation
Source: Biomolecules. 2023 Feb 15;13(2):365. doi: 10.3390/biom13020365 (PMC9953295; doi:10.3390/biom13020365)
Supplement: Supplementary file 1 [file biomolecules-13-00365-s001.zip › biomolecules-2132723-supplementary.pdf]

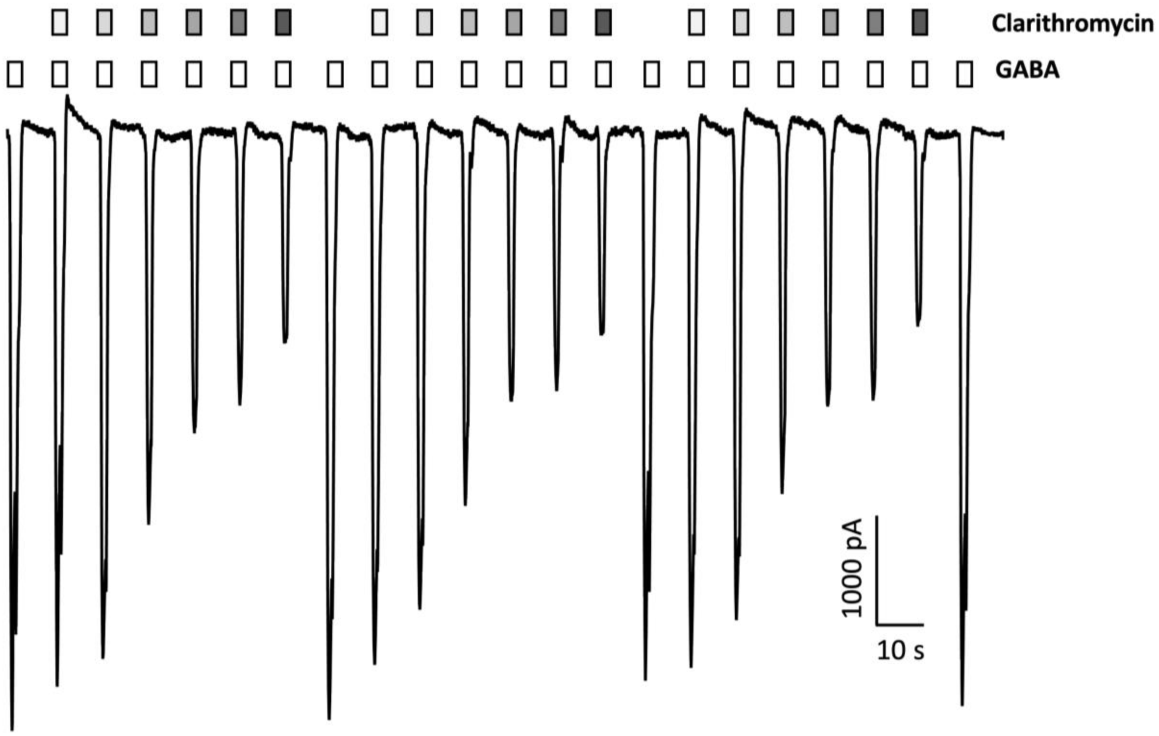

Figure S1. Exemplar Ionflux Mercury current recording. All the high-throughput electrophysiology data collected in this study were recorded using the same 24-episode drug application protocol comprising of 3 repeats of the same ascending presentation of modulator. In every case, the reference agonist was applied prior to co-application with ascending concentrations of modulator, followed by the reference concentration again. The rationale for this approach was to ensure the current responses and modulations were temporally stable, reversible and not use dependent or prone to run down. Figure S1 shoes the effect of co-application of EC_{10} GABA with 1, 3, 10, 30, 100, 300 μM clarithromycin (darker shading indicates increasing clarithromycin concentration). Dataset that were not reproducible (>20% difference between repeated measures) were excluded from our analysis.

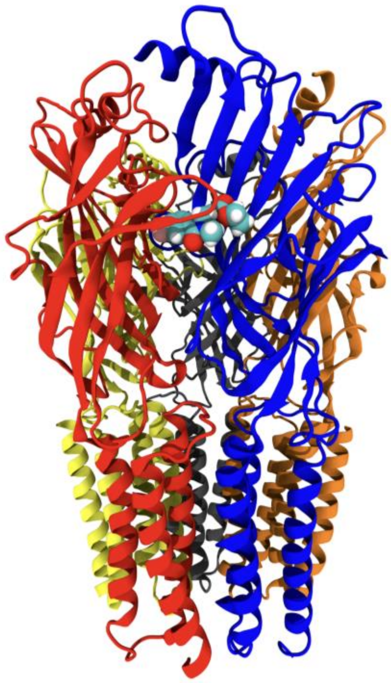

Figure S2. Predicted binding pose for flumazenil. Flumazenil preferentially docked to the canonical benzodiazepine binding site, defined by the α (Loops A, B & C) and γ (loops D & F) subunits. Color key: α subunit (red & grey), β subunit (yellow & orange), γ subunit (blue).

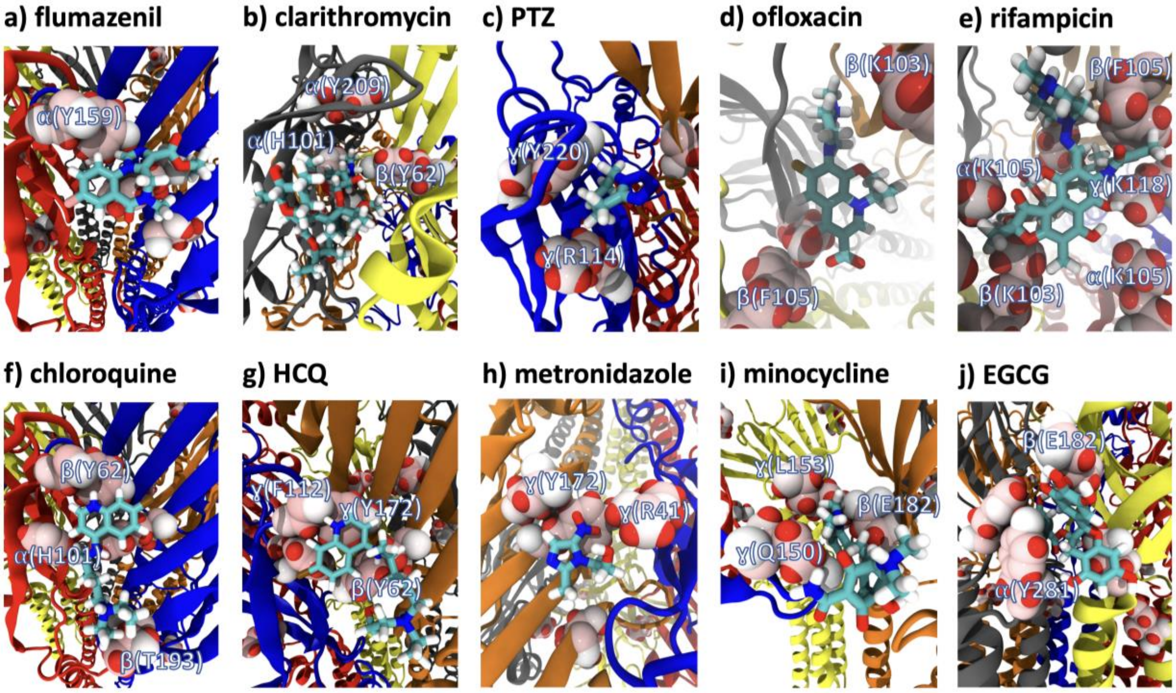

Figure S3. Detailed view of predicted binding poses for 10 compounds studied. Modulators are drawn in stick representation while key interacting residues are shown as CPK spheres. Color key: α subunit (red & grey), β subunit (yellow & orange), γ subunit (blue).
